# Role of adenosine A_3_ receptor and endothelial nitric oxide synthase in patients with traumatic hemorrhagic shock

**DOI:** 10.1007/s00068-025-02853-3

**Published:** 2025-04-08

**Authors:** Yasin Öztaş, Yusuf Ertuğrul Aslan, Elif Funda Şener, Halime Dana, Emre Tuğhan, Nurullah Günay, Abdullah Tuncay Demiryürek

**Affiliations:** 1 Department of Emergency Medicine, Faculty of Medicine, Erciyes University, Kayseri, 38039 Turkey; 2 Department of Medical Biology, Faculty of Medicine, Erciyes University, Kayseri, 38039 Turkey; 3 Erciyes University Genome and Stem Cell Center, Kayseri, 38280 Turkey; 4 Department of Medical Pharmacology, Faculty of Medicine, Gaziantep University, Gaziantep, 27310 Turkey

**Keywords:** Multiple trauma, Hemorrhagic shock, Adenosine, Nitric oxide synthase

## Abstract

**Background:**

The aim of this research is to access the expression of adenosine A3 receptor *(ADORA3)* and nitric oxide synthase 3 *(NOS3)* genes and serum levels of ADORA3 and NOS3 in patients with multiple trauma with hemorrhagic shock.

**Materials and methods:**

The study was performed at Erciyes University between November 2022 and March 2024, in a prospective and controlled manner. Patients diagnosed with traumatic hemorrhagic shock and requiring transfusion in the emergency department were selected as the patients group. Gene expressions were analyzed using quantitative real-time PCR analysis in total RNA samples and serum levels of NOS3 and ADORA3 were detected using ELISA measurements.

**Results:**

In patients with multiple trauma, adenosine A3 receptor *(ADORA3)* gene expression showed a significant increase at discharge when compared to healthy controls (*P* < 0.05). However, serum levels of ADORA3 showed significant decreases at all stages (i.e. at admission, at 24 h, and at discharge) of patients. Although no significant changes were detected in *NOS3* gene expression, marked decreases in serum NOS3 levels were observed at admission and at 24 h in multiple trauma patients (*P* < 0.05). *ADORA3* and *NOS3* gene expressions were found to be significantly diminished in nonsurvivors.

**Conclusion:**

The study emphasizes the importance of *ADORA3* and *NOS3* gene expressions in influencing shock progression in multiple trauma patients. The increase in *ADORA3* gene expression may play a role in restoring vascular reactivity after traumatic shock. Decreased serum NOS3 and ADORA3 levels can contribute to the shock progression in the pathophysiology of multiple trauma.

## Introduction

Polytrauma is a clinical presentation of severe injuries affecting two or more anatomical sites (Abbreviated Injury Scale [AIS] score ≥ 3) with specific physiological impairments. These physiological criteria are considered essential indicators that increase the severity of the injury and the risk of death [1]. A severely injured patient is always at high risk of developing shock, which can quickly lead to death. Even if the shock is adequately managed, polytraumatized patients remain at the risk for the development of a systemic inflammatory response syndrome (SIRS) at the early stages, with the possibility of subsequent multiple organ failure (MOF) [2]. A perilous manifestation of SIRS is coagulopathy, which consequently leads to diffuse hemorrhage. This hemorrhage is surgically untreatable and may hasten the patient back to a state of shock. Approximately 12% of multiple trauma patients are affected by a hemorrhagic shock (HS) that can subsequently lead to organ hypoperfusion and brain damage, being responsible for 50% of all trauma-related deaths within the first 48 h [3]. The brain reacts sensitively to reduced oxygenation and perfusion; in the worst case, this might lead to global cerebral ischemia [4].

Trauma with HS (T/HS) is a medical emergency and leads to MOF that requires rapid intervention. It causes insufficient perfusion of essential tissues and organs due to the decreased blood volume [5]. Recent evidence has shown that overall about 30–50% of patients suffering from T/HS develop MOF [6, 7]. Following T/HS, the traditional guideline-supported therapeutic approach consists of immediate fluid resuscitation and optimized ventilation [8]. Modern resuscitation practice (balanced resuscitation) has limited sanguineous fluids and thus decreased ARDS and MOF. Current research indicates that hypotensive resuscitation is markedly more effective than conventional resuscitation for MOF, SIRS, and mortality [9]. Despite being an ongoing research in trauma, the mechanisms driving this pathology remain largely unknown.

Adenosine triphosphate (ATP) is the universal intracellular energy source and an essential extracellular signaling molecule. Adenosine, an endogenous nucleotide generated from the degradation of ATP, is known to be released during T/HS [10, 11]. Additionally, equilibrative nucleoside transporters (ENTs) can directly release adenosine into the extracellular fluid [12, 13]. Adenosine acts through binding to four G protein-coupled adenosine receptors (A1R, A2a, A2b, A3R) [14, 15]. Cellular stress, hypoxia, or inflammation activate it when adenosine levels rise. Adenosine A3 receptor (ADORA3) suppresses inflammation by acting specifically on inflammatory cells (e.g., mast cells, macrophages). Some cancer cells have shown ADORA3 to mediate apoptosis induction. It is also known to limit ischemia/reperfusion injury by showing cardioprotective effects [16].

Endothelial nitric oxide (NO) is a critical mediator of proper vascular function [17]. Endothelial NO production by eNOS (NOS3) has consistently been considered neuroprotective [18–20]. After subarachnoid hemorrhage (SAH), NO concentration declines rapidly [21]. Early local depletion of NO has been identified to be involved in the development of post-ischemic [22] and post-traumatic [23] brain damage. As for SAH, an early reduction of the NO concentration has been observed in patients [24, 25]. This early NO depletion is associated with microcirculatory dysfunction, such as the formation of microarterial constriction [26]. At later time points, the lack of NO was connected to generating (angiographic) vasospasm [27, 28]. NO lowers blood pressure by causing relaxation of vascular smooth muscles. It reduces the risk of thrombosis by inhibiting platelet aggregation. It also improves endothelial cell function and reduces vascular inflammation, thereby preventing the development of atherosclerosis [29]. ADORA3 and NOS3 work together to keep blood vessels healthy and inflammation under control. This makes them very important to study in conditions like hypoxia, ischemia, and chronic inflammatory diseases. This interplay emphasizes their potential roles in cardiovascular and immune system-related disorders [30, 31].

The expression of adenosine A3 receptor (ADORA3) and nitric oxide synthase 3 (NOS3) genes and serum ADORA3 and NOS3 levels in patients with multiple T/HS are unknown. This research was undertaken to investigate the role of both ADORA3 and NOS3 in patients with T/HS.

## Methods

### Study populations

A total of 32 patients with multiple major trauma and hemorrhagic shock who were admitted to the Emergency Department of the Erciyes University Hospital between November 2022 and March 2024 were enrolled in this study. Additionally, 23 healthy subjects selected from hospital staff and their families were included as age- and sex-matched controls. The local ethics committee approved the study protocol (Decision number: 2022/716, and date: 26.10.2022), and informed consent was obtained from all participants. The present research was conducted according to the principles of the Declaration of Helsinki. All patients received the standard therapy as needed. Central venous pressure (CVP) and the mean arterial pressure (MAP) were followed in all subjects.

Patients included in the study met the criteria for multiple trauma, defined as an AIS score ≥ 3 in at least two different anatomical regions. Additionally, all patients presented with hemorrhagic shock, characterized by active bleeding requiring blood transfusion and at least one of the following clinical or laboratory findings: hemodynamic instability requiring resuscitation, a shock index (heart rate/systolic blood pressure) ≥ 1.0, serum lactate level ≥ 4 mmol/L, or a base deficit ≤-6.0 mmol/L. Only patients aged 18 years or older were included in the study. We did not apply an injury severity score (ISS) [32] cut-off because our primary focus was on patients with hemorrhagic shock requiring transfusion, which inherently indicates a high injury burden. ISS was retrospectively calculated based on all computed tomography (CT) findings. Two emergency medicine specialists performed the scoring independently, and discrepancies were resolved by consensus. The ISS calculation followed the guidelines of the AIS, using the most severe injuries in different body regions to determine the final score.

Patients who experienced immediate mortality in the emergency department were excluded from the study to ensure the analysis focused on individuals who survived the initial resuscitation period. Patients with chronic diseases (e.g., hypertension, diabetes, renal failure), those on long-term medication, and pregnant or breastfeeding women were excluded to minimize potential confounding effects on ADORA3 and NOS3 expression and serum levels. The prehospital health services referred all patients in this research to the emergency department. In Turkiye, prehospital health services cannot give blood products to patients. Prehospital health services present the patient to the emergency department within 20 min of receiving the patient. T/HS patients can receive only crystalloid solution (up to 1000 ml) before admission to the emergency department.

In the patient group, 2 ml of venous blood was collected in EDTA tubes at three different time points (admission, 24th hour, and discharge), while in the control group, 2 ml of venous blood was collected once. Blood samples were taken at admission without treatment in the emergency department, except for crystalloids given by prehospital health services. After the blood samples were taken at admission, all patients were administered blood product transfusions and crystalloids.

### Blood samples

Fasting peripheral blood samples were collected in 2 ml vacutainer blood collection tubes. Blood specimens were allowed to clot for 30 min and then serum was separated by cold (at 4 °C) centrifugation at 4000 g for 20 min. Collected serums were transferred into plain tubes and kept at − 80 °C until analysis. These samples were used for the determination of serum NOS3 and ADORA3 protein levels by ELISA analysis.

### Gene expression studies

As described previously, 2 ml of blood samples were collected into sterile vacuum tubes containing EDTA from all participants for evaluating mRNA expression [33]. The TRIzol-based extraction method was used to isolate total RNA from all of the study participants. Concentrations of RNAs were then measured using BioSpec-Nano (Shimadzu). Samples with 260/230 values below 2 were excluded from the study. RNA samples were stored at -80 ◦ C until use. Transcriptor High Fidelity cDNA Synthesis Kit (Roche) was used for cDNA synthesis. NOS3 and ADORA3 gene expressions were determined by quantitative real-time PCR with a LightCycler 480 system (Roche Diagnostics). GAPDH was utilized as the endogenous reference. cDNA was amplified using the Roche LightCycler 480 real-time PCR system at 95 °C for 10 s, 60 °C for 30 s, and 72 °C for 60 s for 45 cycles, after initial denaturation at 95 °C for 10 min. All samples were prepared twice and each preparation set up in triplicate. Results were analyzed using the 2^−ΔΔCt^ method, according to the formula: ΔΔC_t_ = ΔC_tNOS3 or ADORA3_ − ΔC_tACTB_, where C_t_= threshold cycle.

### ELISA measurements

Serum levels of NOS3 and ADORA3 were determined using ELISA kits (201-12-0920, Human NOS3/eNOS-3, Nitric oxide synthase, endothelial, ELISA Kit, and 201-12-6638, Human A3/ADORA3, Adenosine A3 Receptor, ELISA Kit, Sunred, China). All absorbances were detected at 450 nm using a microplate reader (Promega Glumax Multi Detection System). All values were expressed in ng/ml. All ELISA measurements were carried out in a blinded manner.

Serum levels of NOS3 and ADORA3 were determined using ELISA kits (EH0554, Human NOS3/eNOS, Nitric oxide synthase, endothelial, ELISA Kit, and EH4067, Human A3/ADORA3, Adenosine A3 Receptor, ELISA Kit, FineTest, Wuhan Fine Biotech Co., Ltd., Wuhan, Hubei, China). All absorbances were detected at 450 nm using a microplate reader (Epoch Microplate Spectrophotometer, BioTek Instruments, Winooski, VT, USA). All ELISA measurements were carried out in a blinded manner, and the Gen5 analysis program was used in order to calculate the concentrations.

### Statistical analysis

SPSS 25.0 (Statistical Package for the Social Sciences) or GraphPad Instat (version 3.05, GraphPad Software Inc., San Diego, CA, USA) program were utilized for statistical analysis. The suitability of the data for normal distribution was determined using the Kolmogorov- Smirnov test. Continuous variables with normal distribution are expressed as mean ± standard deviation or standard error and categorical variables as number (n) and percentage (%). ANOVA followed by Dunnett test was applied to compare normally distributed continuous data between groups. Kruskal-Wallis followed by Dunn test was utilized to compare abnormally distributed data between groups. Trauma type such as blunt or penetrating has been added as the covariate to the statistical analysis. The unpaired Student’s t test was used to compare the differences between mean values of two groups. Chi Square test was used for comparisons between categorical data between two groups. Pearson or Spearman tests were used to identify the correlations. Any value of P which is 0.05 or less is considered significant.

## Results

A total of 32 patients with traumatic hemorrhagic shock and 23 healthy volunteers were included in this study. The laboratory, clinical, and demographic characteristics and distribution of injury regions of the groups are given in Table 1. Compared with the controls, gender, the average age, blood urea nitrogen, creatinine, total bilirubin, hemoglobin and platelet counts in patients group were similar. Although respiratory and pulse rates, glucose, aspartate aminotransferase, and alanine aminotransferase levels were increased, temperature, systolic and diastolic blood pressure were detected to be decreased in the patients group when compared to the controls (Table 1). Injury severity score for blunt trauma was significantly higher than score for the penetrating type of trauma (*P* < 0.001). All patients had injuries to at least two different body regions. The highest incidence of injuries was found to be chest injuries (27%), followed by extremity fractures (18%), spinal cord injuries (18%), abdominal trauma (16%), traumatic head injuries (11%), pelvic fractures (10%), and genitourinary injuries (6%).

**Table 1:** Demographic, clinical, laboratory characteristics and distribution of injury regions of the study cases

Parameters	Patients (*n* = 32)	Controls (*n* = 23)	*P*
Age (year)	34.19 ± 16.69	31.09 ± 12.15	0.4552
Gender, n (%)			
Male	24 (75.0)	17 (75.0)	
Female	8 (25.0)	6 (25.0)	0.9273
Temperature (℃)	35.91 ± 0.29	37.23 ± 0.65	**0.0464**
Respiratory rate (beats/min)	22.94 ± 4.45	19.42 ± 7.41	**0.0325**
Pulse rate (beats/min)	113.03 ± 15.58	91.25 ± 11.52	**< 0.0001**
Systolic BP (mm Hg)	103.53 ± 20.85	121.19 ± 10.93	**0.0005**
Diastolic BP (mm Hg)	62.81 ± 16.86	76.42 ± 10.34	**0.0012**
Saturation (%)	94.47 ± 4.42	-	
Shock index	1.12 ± 0.26	-	
GCS	9.59 ± 5.02	-	
Trauma type			
Blunt	23 (71.9)	-	
Penetrating	9 (28.1)	-	
ISS of all patients	51.03 ± 15.88	-	
Red Blood Cell Transfusion in the ED (unit)	1.94 ± 1.19		
pH	7.28 ± 0.14	-	
Lactate (mmol/L)	4.61 ± 3.33	-	
Base deficit (mmol/L)	-8.38 ± 5.73	-	
Glucose (mg/dL)	174.92 ± 79.88	107.97 ± 16.83	**0.0002**
BUN (mg/dL)	25.82 ± 35.08	15.95 ± 9.91	0.1961
Creatinine (mg/dL)	1.25 ± 1.03	1.01 ± 0.64	0.3279
Na^+^ (mmol/L)	138.28 ± 5.67	-	
K^+^ (mmol/L)	4.01 ± 0.74	-	
Cl^−^ (mmol/L)	102.88 ± 6.14	-	
AST (U/L)	164.76 ± 92.63	21.22 ± 10.91	**< 0.0001**
ALT (U/L)	107.08 ± 94.32	22.45 ± 12.31	**< 0.0001**
Total bilirubin (mg/dL)	0.36 ± 0.17	0.41 ± 0.11	0.2222
INR	1.08 ± 0.17	-	
Hemoglobin (g/dL)	13.26 ± 2.47	13.01 ± 3.14	0.7424
Platelet (10^3^/µL)	277.72 ± 65.40	276.21 ± 46.43	0.9248
Arterial pO_2_ (mm Hg)	104 ± 30.99	-	
Arterial pCO_2_ (mm Hg)	33.93 ± 9.63	-	
Distribution of injury regions, n (%)			
Traumatic brain injury	11 (34)	-	
Thorax injury	27 (84)	-	
Abdominal injury	16 (50)	-	
Pelvic injury	10 (31)	-	
Genitourinary injury	6 (19)	-	
Extremity injuries	18 (56)	-	
Spinal injury	18 (56)	-	

All results were parametric and are presented as mean (SD) except gender which is shown as n (%); BP, Blood pressure; BUN, Blood Urea Nitrogen; AST, aspartate aminotransferase; ALT, alanine aminotransferase; INR, International normalized ratio; GCS, Glasgow Coma Scale; ISS, injury severity score; ED, emegency department

All patients underwent a computed tomography. Direct X-ray (44%), ultrasonography (25%) and magnetic resonance imaging (3%) techniques were also applied to the patients. All patients received red blood cell transfusion and physiological saline in the emergency department. Eight patients (25%) received blood product transfusions within the first 24 h after hospitalization. Six patients (18.8%) received red blood cell transfusions, three patients (9.4%) received fresh frozen plasma, two patients (6.3%) received platelet transfusion, and two patients (6.3%) received fibrinogen. Other medical applications included tranexamic acid (16%) and antibiotic therapy (53%). There was no mortality at first 24-hour of admission. However, mortality was detected in 7 patients (21%) before discharge. The demographic and clinical characteristics of the patients, including trauma mechanisms and AIS scores by body region, are summarized in Table 2.

**Table 2:** Comparison of patient characteristics, injury severity, and AIS scores by trauma mechanism

	Blunt Trauma (*n*:23)	Penetrating Trauma (*n*:9)	*P*
Age, median (IQR)	28 (29)	33 (23)	0.644
Gender, n (%) Male Female	15 (65.2) 8 (34.8)	9 (100) 0 (0)	0.070
ISS, median (IQR)	57 (18)	29 (17)	**< 0.001**
AIS, median (IQR) Head and Neck Maxillofacial Thorax Abdomen Extremities External	2 (4) 2 (3) 4 (1) 4 (3) 3 (3) 1 (1)	0 (1) 0 (1) 4 (5) 3 (4) 1 (5) 1 (3)	**0.001** **0.002** 0.109 0.064 0.276 0.584
Red Blood Cell Transfusion in the ED (unit), median (IQR)	2 (2)	1 (1)	0.164
In-hospital mortality, n (%)	6 (26.1)	1 (11.1)	0.640

ISS, injury severity score; AIS, abbreviated injury score; IQR, interquartile range; ED, emergency department

**Fig. 1:**
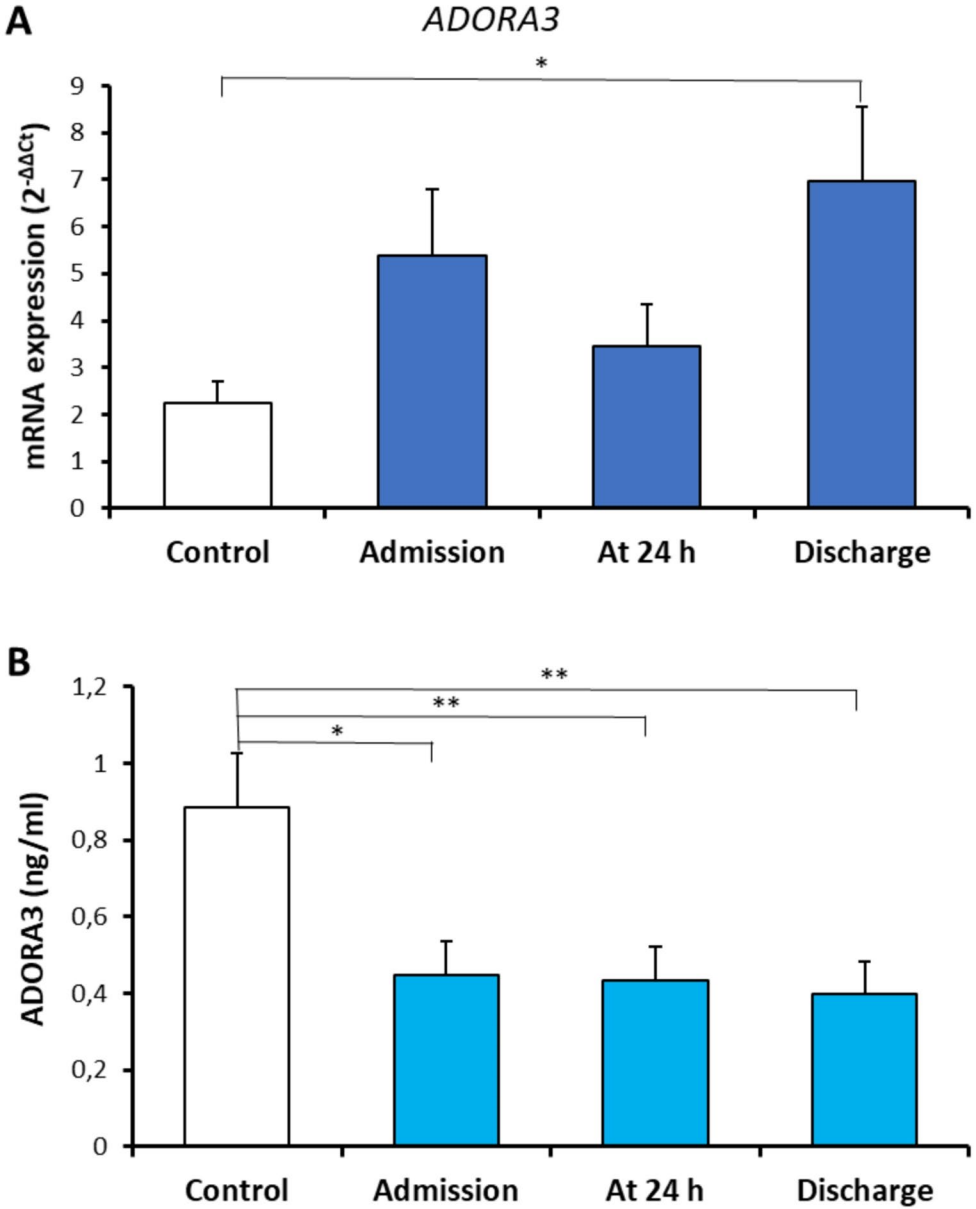
Comparison of the peripheral blood ADORA3 mRNA expression (**A**), serum ADORA3 levels (**B**) in healthy controls (*n* = 23, open bars) and in patients with traumatic hemorrhagic shock (*n* = 32, colored solid bars). Values are given as mean ± SEM. *P < 0.05, **P < 0.01

Comparison of the peripheral blood ADORA3 mRNA expression and serum ADORA3 levels in healthy controls and in patients with traumatic hemorrhagic shock are shown in Fig. 1. There was a marked increase in ADORA3 mRNA expression at discharge. However, marked depressions in serum ADORA3 levels were detected in patients at admission, at 24 h, and at discharge (Fig. 1).

**Fig. 2:**
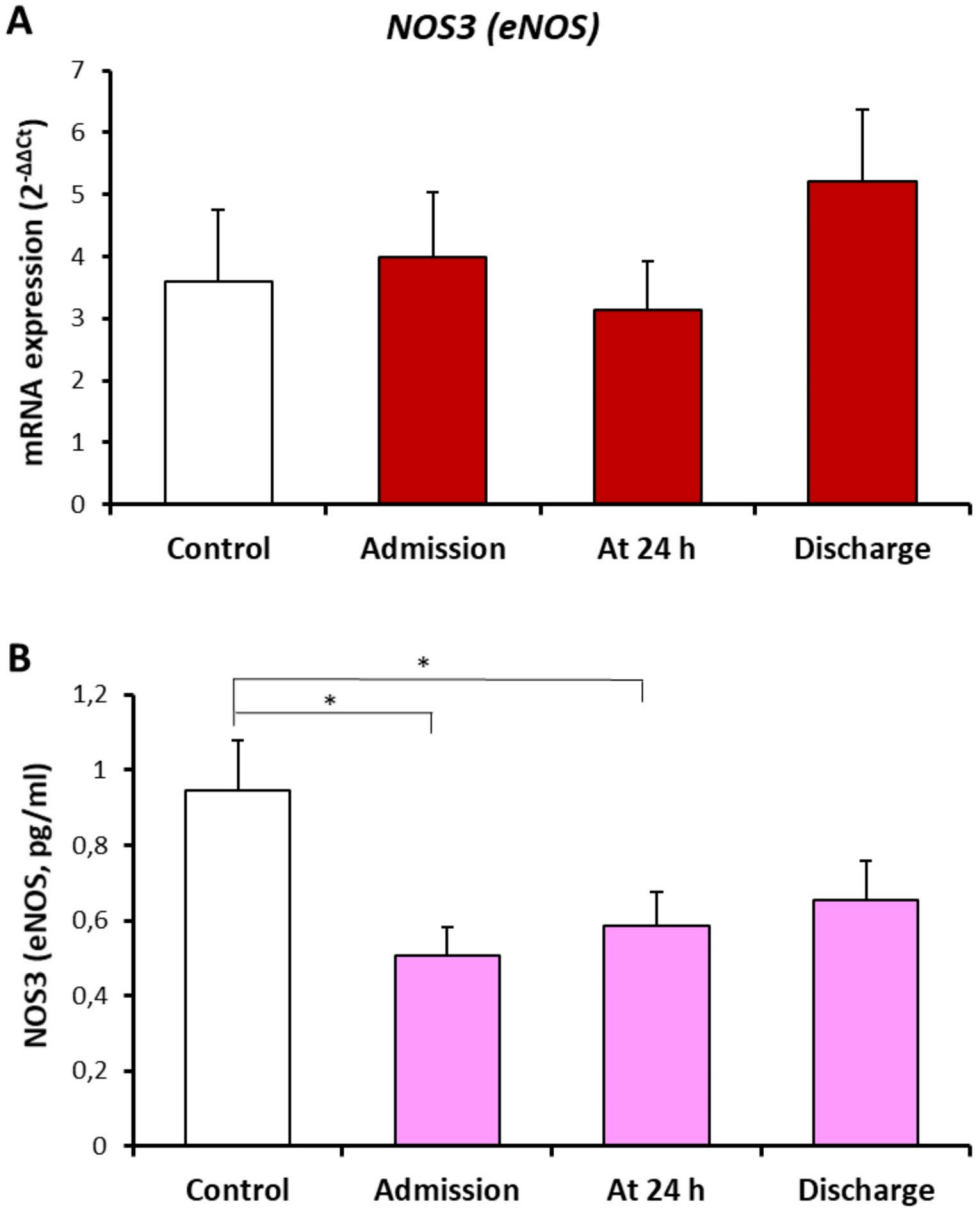
Comparison of the peripheral blood NOS3 mRNA expression (A), serum NOS3 levels (B) in healthy controls (*n* = 23, open bars) and in patients with traumatic hemorrhagic shock (*n* = 32, colored solid bars). Values are given as mean ± SEM. *P < 0.05

**Fig. 3:**
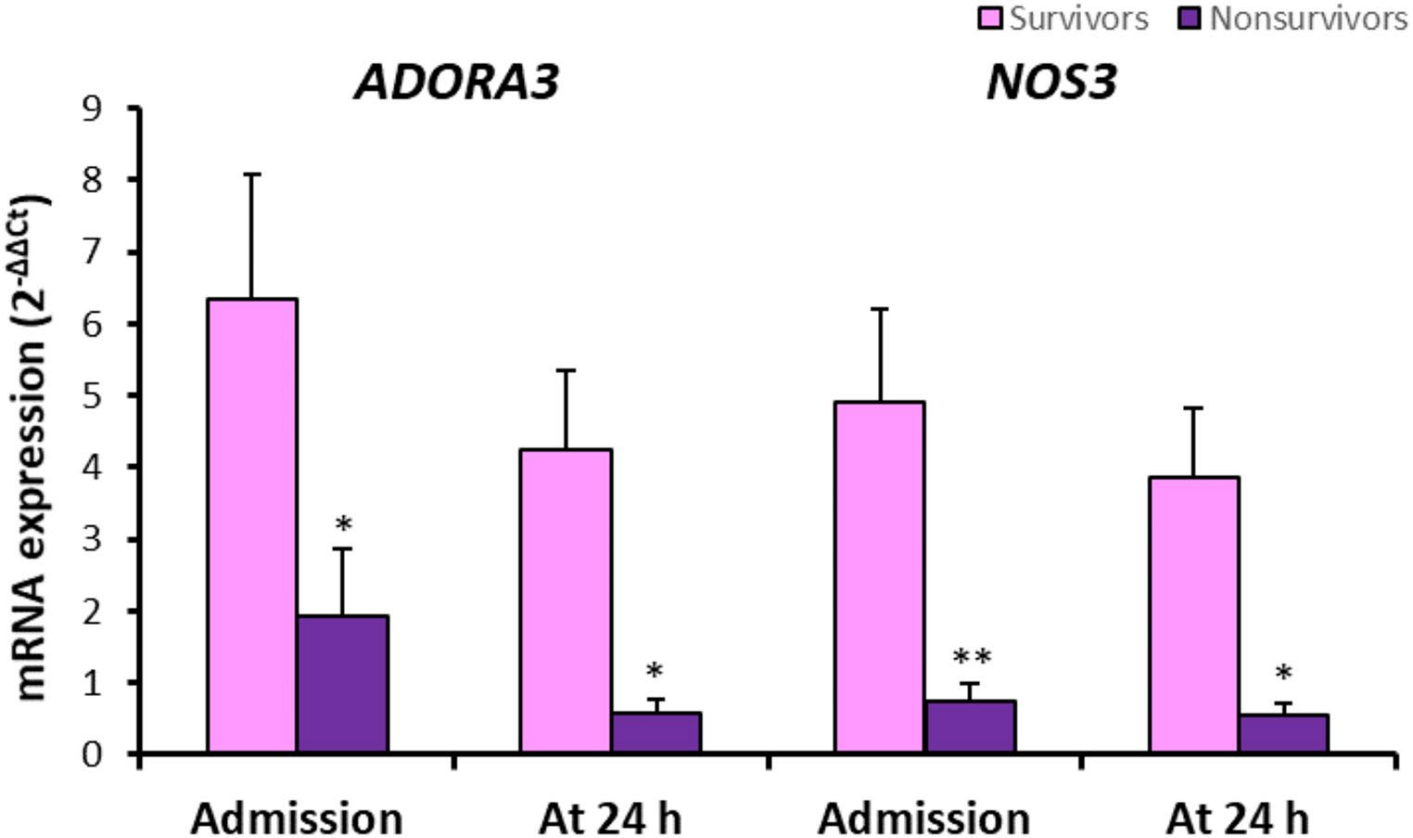
Comparison of the peripheral blood ADORA3 and NOS3 mRNA expressions in patients with survivors (*n* = 25) and nonsurvivors (*n* = 7). Values are given as mean ± SEM. *P < 0.05, **P < 0.01

Peripheral blood NOS3 mRNA expression and serum NOS3 levels in healthy controls and in patients with traumatic hemorrhagic shock are shown in Fig. 2. Although there was no. marked changes in NOS3 mRNA expression, marked reductions in serum NOS3 levels were measured in patients at admission and at 24 h (Fig. 2). When comparing survival and mortal patients, gene expressions for both ADORA3 and NOS3 were found to be significantly decreased in the event of mortality (Fig. 3).

Correlation analysis revealed that there were positive correlations between ADORA3 and NOS3 mRNA expressions at all stages (i.e. at admission, at 24 h, and at discharge) of patients (Table 3). There was a negative correlation between NOS3 mRNA expression and serum NOS3 level during admission period. Another negative correlation was noted between serum ADORA3 level and ADORA3 mRNA expression at 24 h (Table 3).

**Table 3:** Significant correlations between the measured parameters of the groups

Parameters	Correlation			Coefficient of		*P*
	coefficient (*r*)			determination (*r*^2^)		
*Admission*:						
*ADORA3* mRNA ↔ *NOS3* mRNA	0.7583			0.5750		< 0.0001
Serum NOS3 level ↔ *NOS3* mRNA	-0.4503			0.2028		0.0273
*At 24 h*:						
*ADORA3* mRNA ↔ *NOS3* mRNA	0.8398			0.7052		< 0.0001
*ADORA3* mRNA ↔ Serum ADORA3 level	-0.4401			0.1937		0.0314
Serum ADORA3 level ↔ ISS	0.568			0.3226		0.007
*ADORA3* mRNA *↔* ISS	-0.484			0.2342		0.005
*Discharge*:						
*ADORA3* mRNA *↔ NOS3* mRNA	0.9145			0.8363		< 0.0001
Serum NOS3 level ↔ ISS	0.614			0.3769		0.003
**Penetrating Trauma**						
*At 24 h*:						
*ADORA3* mRNA *↔ NOS3* mRNA	0.833			0.6938		0.005
*ADORA3* mRNA *↔* ISS	-0.840			0.7056		0.005
Serum ADORA3 level ↔ ISS	0.916			0.8390		0.004
*Discharge*:						
Serum NOS3 level ↔ ISS	0.947			0.8968		0.001
*ADORA3* mRNA *↔ NOS3* mRNA	0.993			0.9860		< 0.001
**Blunt Trauma**						
*Admission*:						
*ADORA3* mRNA ↔ *NOS3* mRNA	0.815			0.6642		< 0.001
Serum NOS3 level ↔ *NOS3* mRNA	-0.596			0.3552		0.025
Serum ADORA3 level ↔ Serum NOS3 level	0.899			0.8082		< 0.001
*At 24 h*:						
Serum ADORA3 level ↔ Serum NOS3 level	0.820			0.6724		< 0.001
*ADORA3* mRNA ↔ *NOS3* mRNA	0.885			0.7832		< 0.001
*Discharge*:						
Serum NOS3 level ↔ ISS	0.659			0.4342		0.010
Serum ADORA3 level ↔ Serum NOS3 level	0.797			0.6352		< 0.001
*ADORA3* mRNA ↔ *NOS3* mRNA	0.847			0.7174		< 0.001

We also performed a correlation analysis between the trauma type and the expressions of ADORA3 or NOS3. We found a strong correlation between the levels of ADORA3 mRNA and NOS3 mRNA at 24 h and at the time of discharge, no matter what kind of trauma it was. However, at admission, this correlation was significant in blunt trauma but not in penetrating trauma (Table 3).

Correlation analysis between trauma type and ADORA3 or NOS3 serum levels revealed that in blunt trauma, serum ADORA3 and NOS3 levels exhibited a significant positive correlation at all time points. However, this correlation was not observed in penetrating trauma (Table 3).

## Discussion

The present study showed that serum ADORA3 and NOS3 levels were significantly reduced in patients with multiple trauma with hemorrhagic shock. We have detected an increase in *ADORA3* gene expression at discharge in patients, but there were no changes in *NOS3* gene expressions. On the other hand, depressed *ADORA3* and *NOS3* gene expressions were evident in the nonsurvivors compared to survivors. Positive correlations between *ADORA3* and *NOS3* mRNA expressions were also observed in patients. The timing of blood sampling revealed critical insights into the progression of shock and the response to resuscitation. Marked decreases in serum ADORA3 levels were seen at admission and after 24 h. These reductions could be due to acute vascular and inflammatory stress. The rise in gene expression during discharge was likely related to normalizing reduced ADORA3 levels. The decreases in NOS3 and ADORA3 levels could be related to therapeutic interventions like blood transfusions and balanced fluid resuscitation. These data underline the importance of starting resuscitation as soon as possible for T/HS patients.

Mild hypothermia was noted in our patients. Hypothermia is a prevalent event in trauma patients. Clinical experiences show that hypothermia is one major cause of severe posttraumatic complications [34]. Hypothermia coupled with multiple trauma is critical since it has been demonstrated that hypothermia when combined with coagulopathy and acidosis, which is recognized as a lethal triad, can worsen the prognosis [35].

This is the first study to evaluate the serum NOS3 levels in patients with multiple trauma with hemorrhagic shock. We have observed suppressed serum NOS3 levels, but the role of NO in traumatic or hemorrhagic shock is controversial. Both elevated [36–38] and depressed [39, 40] serum NO levels have been reported. It has been shown that NO overproduction starts immediately after trauma and returns to normal within 24 h [36]. Beitl et al. [38] suggested that augmented serum NO and blood lactate in patients with polytrauma are markers of serious clinical course, while normal NO levels in conjunction with a very high lactate may signal a fatal prognosis. This study reported that serum NO levels drops to normal values within 12 h [38]. Another study demonstrated that NO levels were higher in patients with multiple bone fractures than in those with femoral fractures, and this may be caused by eNOS induced by mechanical shearing forces [37]. Corbett et al. [41] indicated that eNOS was elevated in the bone tissue in the early phases of fracture healing to adjust the blood flow, and this was critical for fracture healing. On the other hand, low serum nitrite/nitrate levels were detected in trauma patients and stayed low even in the presence of sepsis [39, 40]. Clark et al. [42] showed no difference over time in plasma NO levels detected, but there were rises in cerebrospinal fluid NO levels, which peaked at 30 to 42 h after severe closed-head injury. No differences in posttraumatic plasma NO levels were observed in survivors compared with nonsurvivors [42].

There were no changes in *NOS3* gene expressions in our study. This result consistent with the results presented by Gahm et al. [43] who reported that no detectable difference in the endothelial NOS expression was observed for contusional brain trauma patients, compared with control subjects. On the other hand, iNOS expression was shown to be up-regulated in a time-dependent manner in human brain tissue following contusional trauma [43].

We found for the first time that serum ADORA3 levels were markedly diminished in patients with multiple traumas with hemorrhagic shock. We also showed an increase in *ADORA3* gene expression at discharge in patients. Our results align with the finding showing that A3 receptor expression on the surface of polymorphonuclear neutrophils has been markedly higher in patients with traumatic brain injury, and the level of expression correlated with the severity of injury [44]. The upregulation of *ADORA3* gene expression can restore vascular reactivity after traumatic shock. Elevating endogenous adenosine mitigated the destruction of the alveolar-capillary membrane and decreased lung edema, causing oxygenation and prolonged overall survival after experimental blunt chest injury [45]. Extracellular adenosine generates protective effects by promoting cell survival and suppressing inflammation via adenosine receptors [46]. Adenosine A_3_ receptor stimulation decreases ischemic brain injury through suppression of apoptosis in rodents [47]. Studies have shown that adenosine A_3_ receptors could play a significant role in the adenosine-mediated inhibition of TNF-α formation [48]. These results imply that a rise in adenosine levels may induce protective effects against traumatic injury.

Hemolysis can release red blood cell proteins, such as arginase and hemoglobin. Arginase regulates NO levels by competing with NOS for the substrate L-arginine and limits physiologic NO production [49]. Arginase converts the amino acid L-arginine into urea and ornithine. Thus, arginase released from erythrocytes may contribute to the depletion of L-arginine and the subsequent diminution in the NO necessary to maintain organ perfusion [50]. Free hemoglobin reacts with and depletes NO [51]. Thus, NO is crucial for vascular homeostasis, depletion of NO is an important feature for the development of T/HS.

The overall mortality rate of our study is 21%, which looks pretty modest when juxtaposed with previous cohorts exhibiting a similar mean ISS of 51. A significant factor contributing to this is the exclusion of patients who expired in the emergency room, as our study concentrated on those who survived the initial resuscitation phase. Additionally, all included patients received early and aggressive resuscitation, including blood transfusions, by current trauma management guidelines, which may have improved early survival rates. When analyzing trauma mechanisms separately, we observed that mortality was significantly higher in patients with blunt trauma (26.1%) compared to penetrating trauma (11%). Unlike other studies that report a substantial percentage of fatalities occurring within the initial 24 h due to hemorrhagic shock or severe traumatic brain injuries, our observations indicated no mortality during this timeframe. This differential may be ascribed to variations in patient selection criteria, expedited prehospital transport durations, or the general efficacy of initial trauma care in our environment.

Because ADORA3 and NOS3 play such a big part in traumatic hemorrhagic shock, future treatments might focus on changing these pathways to help patients do better. Activating ADORA3 has demonstrated the ability to reduce inflammation and protect tissues. This means that ADORA3 agonists might be worth looking into as possible extra treatments for trauma patients. The stimulation of ADORA3 receptors in animals may lessen ischemic injury and improve vascular reactivity. This could help people who are in hemorrhagic shock. If you change the activity of NOS3 to make more endothelial NO, it might also help the blood flow to smaller blood vessels and make organs work better. If a person is seriously hurt, using NO donors or therapies that target NOS3 could help restore vascular homeostasis and keep multiple organs from failing. Collectively, an adenosine A3 receptor agonist or a NO donor could be added to the treatment regimen as a replacement therapy for the patients with multiple trauma with hemorrhagic shock. However, additional clinical studies are required to assess the safety and effectiveness of these targeted interventions in trauma populations.

This study has several limitations that should be acknowledged. First, the small size of our sample may make it harder to use our results in other situations and make it harder to find more complex links between ADORA3 and NOS3 levels and clinical outcomes. We need larger, multicenter studies to validate our results and investigate potential subgroup differences. Second, although we collected blood samples at admission, prior to sample collection, patients had received varying amounts of crystalloid fluids during prehospital care. The non-standardised volume of crystalloid administration could have influenced baseline biomarker levels, potentially confounding our results. Future research should aim to minimise such variability by standardising prehospital interventions or stratifying patients based on prehospital fluid resuscitation protocols. A key limitation of this study is the absence of a control group consisting of trauma patients without hemorrhagic shock. This makes it difficult to isolate the specific effects of hemorrhagic shock from the broader impact of trauma itself. More research with a control group will be needed to confirm that the changes in ADORA3 and NOS3 are specific to hemorrhagic shock. Our study provides new information about how ADORA3 and NOS3 are involved in trauma-related pathophysiology, but the absence of a control group of trauma patients without hemorrhagic shock suggested that we were not able to distinguish between the influences of trauma itself and the influences of the hemorrhagic shock.

## Conclusion

In summary, our results demonstrated that ADORA3 and NOS3 serum levels and gene expessions are involved in pathophysiology of the multiple T/HS. Our data may help to better understand the mechanisms responsible for the shock progression in the pathophysiology of multiple trauma. Our results suggest that ADORA3 and NOS3 may be involved in the overall inflammatory response linked to traumatic hemorrhagic shock. However, their potential role as specific biomarkers for monitoring shock progression and resuscitation efficacy remains uncertain and requires further validation. The increased ADORA3 levels at discharge may reflect a compensatory vascular response, which warrants additional investigation to determine its therapeutic implications. A lower level of NOS3 was found in the blood during the acute phase of shock. This suggests that decreased NOS3 may reflect the presence of endothelial dysfunction, which is a feature of multiple organ failure. Incorporating NOS3-modulating therapies into resuscitation protocols could potentially mitigate shock-induced vascular injury. The strong correlation observed between ADORA3 and NOS3 gene expressions highlights their potential involvement in vascular homeostasis; however, their precise role in hemorrhagic shock remains to be fully elucidated. Targeting these pathways could pave the way for personalized treatment approaches aimed at improving survival rates for trauma patients. However, further studies are still necessary to unravel their mechanisms of action in T/HS.

## Data Availability

No datasets were generated or analysed during the current study.
